# The non-human primate striatum undergoes marked prolonged remodeling during postnatal development

**DOI:** 10.3389/fncel.2014.00294

**Published:** 2014-09-22

**Authors:** Lee J. Martin, Linda C. Cork

**Affiliations:** ^1^Division of Neuropathology, Department of Pathology, Johns Hopkins University School of MedicineBaltimore, MD, USA; ^2^Pathobiology Graduate Program, Johns Hopkins University School of MedicineBaltimore, MD, USA; ^3^Department of Neuroscience, Johns Hopkins University School of MedicineBaltimore, MD, USA; ^4^Department of Comparative Medicine, Stanford University School of MedicinePalo Alto, CA, USA

**Keywords:** striatal mosaic, striosome, rhesus monkey, infant, autism, epigenetics, leucine-enkephalin, nucleus accumbens

## Abstract

We examined the postnatal ontogeny of the striatum in rhesus monkeys (*Macaca mulatta*) to identify temporal and spatial patterns of histological and chemical maturation. Our goal was to determine whether this forebrain structure is developmentally static or dynamic in postnatal life. Brains from monkeys at 1 day, 1, 4, 6, 9, and 12 months of age (*N* = 12) and adult monkeys (*N* = 4) were analyzed. Nissl staining was used to assess striatal volume, cytoarchitecture, and apoptosis. Immunohistochemistry was used to localize and measure substance P (SP), leucine-enkephalin (LENK), tyrosine hydroxylase (TH), and calbindin D28 (CAL) immunoreactivities. Mature brain to body weight ratio was achieved at 4 months of age, and striatal volume increased from ∼1.2 to ∼1.4 cm^3^ during the first postnatal year. Nissl staining identified, prominently in the caudate nucleus, developmentally persistent discrete cell islands with neuronal densities greater than the surrounding striatal parenchyma (matrix). Losses in neuronal density were observed in island and matrix regions during maturation, and differential developmental programmed cell death was observed in islands and matrix regions. Immunohistochemistry revealed striking changes occurring postnatally in striatal chemical neuroanatomy. At birth, the immature dopaminergic nigrostriatal innervation was characterized by islands enriched in TH-immunoreactive puncta (putative terminals) in the neuropil; TH-enriched islands aligned completely with areas enriched in SP immunoreactivity but low in LENK immunoreactivity. These areas enriched in SP immunoreactivity but low in LENK immunoreactivity were identified as striosome and matrix areas, respectively, because CAL immunoreactivity clearly delineated these territories. SP, LENK, and CAL immunoreactivities appeared as positive neuronal cell bodies, processes, and puncta. The matrix compartment at birth contained relatively low TH-immunoreactive processes and few SP-positive neurons but was densely populated with LENK-immunoreactive neurons. The nucleus accumbens part of the ventral striatum also showed prominent differences in SP, LENK, and CAL immunoreactivities in shell and core territories. During 12 months of postnatal maturation salient changes occurred in neurotransmitter marker localization: TH-positive afferents densely innervated the matrix to exceed levels of immunoreactivity in the striosomes; SP immunoreactivity levels increased in the matrix; and LENK-immunoreactivity levels decreased in the matrix and increased in the striosomes. At 12 months of age, striatal chemoarchitecture was similar qualitatively to adult patterns, but quantitatively different in LENK and SP in caudate, putamen, and nucleus accumbens. This study shows for the first time that the rhesus monkey striatum requires more than 12 months after birth to develop an adult-like pattern of chemical neuroanatomy and that principal neurons within striosomes and matrix have different developmental programs for neuropeptide expression. We conclude that postnatal maturation of the striatal mosaic in primates is not static but, rather, is a protracted and dynamic process that requires many synchronous and compartment-selective changes in afferent innervation and in the expression of genes that regulate neuronal phenotypes.

## INTRODUCTION

The striatum is a subcortical forebrain gray matter compartment of the basal ganglia comprised of neurons with relatively homogeneous morphology (the principal medium-sized spiny neurons) but diverse connections and neurotransmitters (Crittenden and Graybiel, 2011; Haber et al., 2012). The striatum is divided into the caudate nucleus and putamen that comprise the dorsal striatum, and the nucleus accumbens and olfactory tubercle that comprise the ventral striatum (Heimer et al., 1997; Haber et al., 2012). The synaptic interactions within the striatum and interconnected brain regions function in sensory-motor integration, planning and initiation of somatic movements, cognition, and some types of social-emotional and species-typical behaviors (Heimer and Van Hoesen, 2006; Crittenden and Graybiel, 2011; Baez-Mendoza and Schultz, 2013; Shepherd, 2013; Smith et al., 2014). Anatomical principles of organization of the striatum include the differentiation of the dorsal striatum into at least two primary compartments designated as striosomes (patches) and extrastriosomal matrix (Crittenden and Graybiel, 2011), the differentiation of the nucleus accumbens into core and shell regions (Heimer et al., 1997), and the serial and parallel arrangements of input-output connectivity (Alexander et al., 1986; Heimer and Van Hoesen, 2006; Haber et al., 2012). Striosomes in cat, macaque, and human caudate nucleus are organized as complex reticular networks (Groves et al., 1988; Desban et al., 1989; Manley et al., 1994; Mikula et al., 2009); specifically, in cynomolgus monkey a typical striosome segment is ~335 μm in diameter by ~1 mm in length that then can issue collateral channels (Mikula et al., 2009). Neuronal populations in striosomes and matrix are believed to differ with respect to their time of embryogenesis (van der Kooy and Fishell, 1987), afferent and efferent connectivity with cerebral cortex, substantia nigra and globus pallidus (Goldman-Rakic, 1981; Gerfen, 1984, 1985; Gimenez-Amaya and Graybiel, 1991), expression of neurotransmitters or neurotransmitter-related enzymes (Graybiel and Ragsdale, 1978; Gerfen and Young, 1988; Martin et al., 1991a), neurotransmitter receptors (Martin et al., 1993), and intracellular second messenger effector systems (Fotuhi et al., 1993; Goto et al., 1994). However, more recent studies do not support the concept of a dual striosome-matrix striatofugal system in non-human primates (squirrel monkeys) because elegant single-axon tracings show that axons of striosomal neurons target globus pallidus and substantia nigra (Levesque and Parent, 2005).

The development of the neurochemical organization of the basal ganglia in rhesus monkey and rat is vulnerable to early postnatal experience (Martin et al., 1991b; Robbins et al., 1996; Thorsell et al., 2006). The normal compartmental arrangements of peptidergic neurotransmitters of striatal output neurons, the localization of neuropeptides within striatal synaptic targets (globus pallidus and substantia nigra), and the dopaminergic afferents to the striatum from substantia nigra are all profoundly altered in adult rhesus monkeys that experienced severe sensory/social deprivation during their first year of infancy (Martin et al., 1991b). Rhesus monkeys deprived of ample infant-mother and infant-peer interactions during early development manifest psychosocial abnormalities and motor impairments, including withdrawal and fearfulness, lack of play, apathy, learning deficits, stereotypic movements, and self-injurious behaviors; as adults such monkeys do not show appropriate parental behavior and are indifferent and abusive to their offspring (Vicedo, 2009). Because monkeys that experienced social/sensory deprivation have neurochemical alterations in their basal ganglia (Martin et al., 1991b), and because the behavioral disabilities induced by social/sensory deprivation of infants primates persist into adulthood (Vicedo, 2009) and suggest dysfunction of the basal ganglia, we have hypothesized that the normal postnatal development of the structural and functional organization of the basal ganglia, particularly the dorsal striatum, is partly determined by social environment and experience (Martin et al., 1991b). However, the normal maturational events that occur in the striatum of primates during postnatal development must be understood before this theory of experience-dependent neurodevelopment of the basal ganglia can be substantiated, and before the possible cellular and molecular mechanisms responsible for the mutability of basal ganglia organization by early postnatal experience can be understood. This study was initiated to evaluate the temporal and spatial development of striatal cytology and chemical neuroanatomy in normal socially reared infant rhesus monkeys.

## MATERIALS AND METHODS

### MONKEYS

Twelve infant rhesus monkeys (*Macaca mulatta*; **Table 1**) and four adult rhesus monkeys 4–7 years old (two males and two females) were used in this study. The animal protocols employed were approved by the Johns Hopkins University School of Medicine Animal Care and Use Committee. All infant monkeys were reared socially with mothers and peers in a colony and euthanized less than three hours after removal from the colony.

**Table 1:** Infant rhesus monkey used.

Age	*N*	Gender
1 day	2	1 female, 1 male
1 month	2	1 female, 1 male
2 months	2	1 female, 1 male
4 months	2	1 female, 1 male
6 months	1	male
9 months	1	male
12 months	2	1 female, 1 male

### PREPARATION OF BRAINS

The animals were restrained with ketamine, deeply anesthetized with intravenous sodium pentobarbital, weighed, and, after a thoracotomy, perfused intra-aortically with 0.9% saline followed by phosphate-buffered 4% paraformaldehyde. After perfusion, the brains were removed from the skull and were weighed. The brain was placed on its dorsal (superior) surface and cut coronally into 1-cm-thick slabs with the first (most anterior) cut placed at a random start in frontal lobe. The brain slabs were postfixed (2 h) in fixative, rinsed thoroughly in buffer, cryoprotected (overnight) in two changes in phosphate-buffered 20% glycerol, and frozen in isopentane chilled by dry ice.

### HISTOLOGY

The cerebrum from each monkey was cut into serial coronal sections (40 μm) on a sliding microtome. The cutting began anterior to the striatum and continued throughout the entire striatum, including the posterior putamen. Every 10th through 15th section was selected with a random start to give a systematic-uniform set of subsampled sections through the entire cerebrum of each brain for stereological analysis. To visualize gray and white matter, every 10th section was stained with cresyl violet. These sections were used for volumetric analysis of the striatum using point counting and the Cavalieri principle as described (Calhoun et al., 1996; Mouton et al., 1998) and for microscopic examination of the striatal mosaic cytology and developmental cell death by profile counting of apoptotic figures. Cresyl violet staining is very useful for identifying apoptotic cells in nervous tissue (Al-Abdulla et al., 1998; Martin et al., 1999; Northington et al., 2001; Lok and Martin, 2002; Mueller et al., 2005). The other selected sections were processed immunohistochemically using a standard peroxidase anti-peroxidase procedure with diaminobenzidine as chromogen and primary antibodies to tyrosine hydroxylase (TH), substance P (SP), leucine-enkephalin (LENK), and calcium binding protein calbindin-D28 as described (Martin et al., 1991a,b,c). The groups of sections from each monkey were coded for the immunohistochemistry so that the technician was unaware of monkey age. The primary antibodies that were used were mouse monoclonal and rabbit polyclonal that have been characterized previously in primate brain (Martin et al., 1991a,b,c). These antibodies detected SP (Sera Laboratory International, West Sussex, England, and ImmunoStar, Hudson, WI, USA), LENK (AbD Serotech, Raleigh, NC and ImmunoStar), calbindin (Sigma, St. Louis, MO, USA), and TH (Boehringer Mannheim and Eugene Tech International, Allendale, NJ, USA). Variations in the thickness of sections were determined microscopically by measuring in the *z*-axis to be minimal. The scant sections that were thicker or thinner than usual were not included in this analysis. Sections from brains at all of the different developmental periods were matched for level and processed concurrently using aliquots from identical batches of reagents.

### IMAGE ACQUISITION AND ANALYSIS

An image analysis system and Inquiry software (Loats Associates, Westminster, MD, USA) was used to quantify by densitometry the relative levels of immunoreactivities for SP, LENK, TH, and calbindin in the caudate nucleus, putamen, and nucleus accumbens in sections of monkey brain. Digital images of sections containing these regions were acquired under constant low ambient light conditions with a high-resolution, low-light sensitive camera. Densitometric measurements of immunoreactivity specifically within visually identified striosome and matrix regions of caudate nucleus and putamen and core and shell regions of nucleus accumbens were made, by an observer unaware of monkey age, from coded slides using a method described (Ballough et al., 1995) in at least five matched sections containing the striatum from each monkey.

### STATISTICAL ANALYSES

Brain weights, striatal volumes, cell counts, and densitometry measurements were evaluated statistically by one-way ANOVA followed by a Newman–Keuls *post hoc* test.

### PHOTOGRAPHY AND FIGURE CONSTRUCTION

Monkey brain sections were imaged under identical conditions and analyzed using identical parameters. Original images used for figure construction were generated using a photographic print enlarger and digital photography. Low magnification panoramic images were made as hardcopy prints by directly exposing the glass slide with the brain section to photographic print paper using constant enlargement and exposure settings. Digital images were captured as TiF files using a SPOT digital camera and SPOT Advanced software (Diagnostic Instruments) or a Nikon digital camera (DXM1200) and ACT-1 software. Images were altered slightly for brightness and contrast using ArcSoft PhotoStudio 2000 or Adobe Photoshop software without changing the content and actual result. Figure composition was done using CorelDraw software with final figures being converted to TiF files. Files of composite figures were adjusted for brightness and contrast in Adobe Photoshop.

## RESULTS

### BRAIN SIZE AND STRIATAL VOLUME DURING THE 1ST POSTNATAL YEAR

**FIGURE 1:**
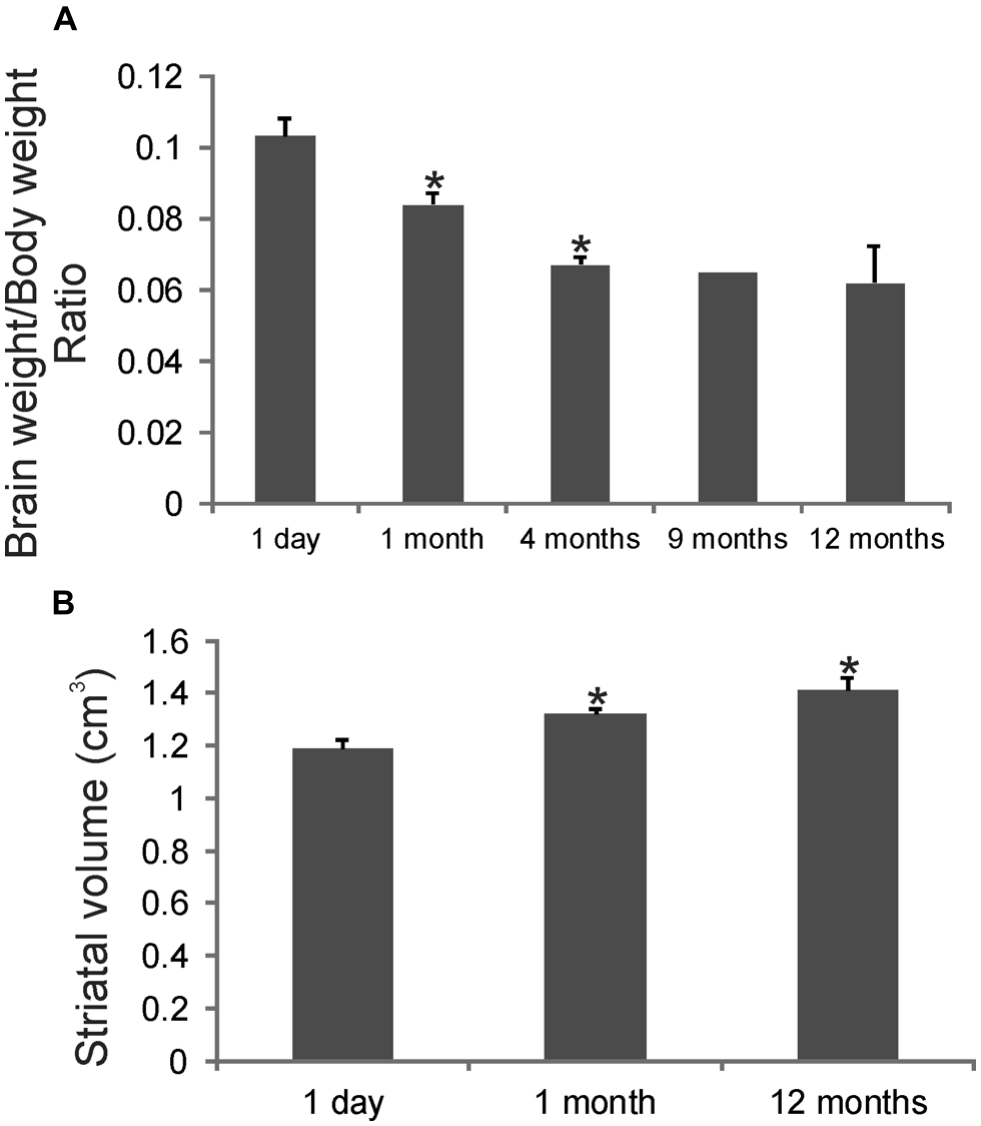
**Gross brain measurements in rhesus monkeys during postnatal development. (A)** Graph showing the brain weight to body weight ratios in rhesus monkeys during the first postnatal year. Values are mean ± SD. Asterisks denote significant difference (*p* < 0.05) from preceding age. **(B)** Stereological determination of striatal volumes in rhesus monkeys at 1 day, 1 and 12 months of age. Asterisks denote significant difference (*p* < 0.05) from 1 day.

Brain weights and striatal volumes were assessed in our cohort of developing rhesus monkeys. Determinations of brain weight to body weight ratios revealed a significant progressive decrease at 1 and 4 months compared to 1 day (**Figure 1A**). The brain to body weight ratios at 4, 9, and 12 months of age were similar. Striatal volume significantly increased progressively during the first year of life (**Figure 1B**). Striatal volume was about 1.2 cm × 1.2 cm × 1.2 cm at 1 day of age (**Figure 1B**). At 1 month of age striatal volume increased significantly compared to 1 day (**Figure 1B**), and then achieved ~1.4 cm × 1.4 cm × 1.4 cm at 12 months of age (**Figure 1B**).

### THE STRIATAL MOSAIC IN INFANT RHESUS MONKEYS AS SEEN BY NISSL STAINING

**FIGURE 2:**
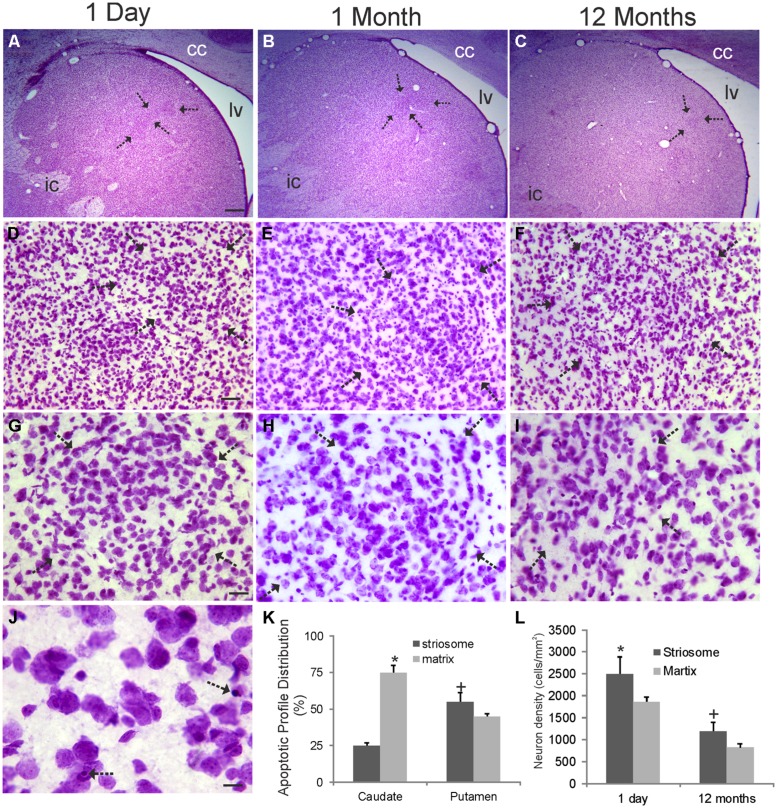
**Cytology of the rhesus monkey caudate nucleus during the first year of postnatal life.** Images shown are from the dorsomedial caudate nucleus of monkeys 1 day, 1 and 12 months of age as seen in coronal sections stained with cresyl violet. Medial is left and top is dorsal. lv, lateral ventricle; cc, corpus callosum; ic, internal capsule. **(A–C)** Inhomogeneities in the cellular organization are seen as discrete cellular islands (arrows) and are most prominent at 1 day of age. **(D–F)** The cellular islands (arrows) appear different from the surrounding striatal parenchymal cells (matrix) because their arrangement and density (see panel **L**) are different. **(G–I)** The cellular islands are separated from the surrounding striatal matrix cells by a cell density-low annulus. This distinction becomes less prominent with maturation **(I)**. **(J)** Programmed cell death by apoptosis (arrows) is observed readily in the infant striatum. **(K)** Graph showing the distribution of apoptotic cells in the caudate nucleus and putamen in striosome (island) and matrix compartments in monkeys 1 day of age. Values are mean ± SE . Matrix cell apoptosis was significantly higher (asterisk, *p* < 0.001) than striosome apoptosis in caudate, while striosome cell apoptosis was significantly higher (+, *p* < 0.01) than matrix apoptosis in putamen. **(L)** Cell densities in striosome (island) and matrix compartment in the caudate in monkeys at 1 day and 12 months of age. Values are mean ± SD. Symbols (* and +) denote significant difference (*p* < 0.05) from matrix. Scales bars: 625 μm **(A,** same for **B,C)**; 45 μm **(D,** same for **E,F)**; 22.5 μm **(G,** same for **H,I)**; 8 μm **(J)**.

Cresyl violet-stained sections through the rhesus monkey striatum at 1 day, 1, and 12 months of age are shown in **Figure 2**. Islands of cells in the caudate nucleus were seen at all ages at low magnification (**Figures 2A–C**). These cellular islands are visible in Nissl sections because the arrangement of cells within them appears as a distortion against the background arrangement of matrix cells that comprise the majority of the striatal parenchyma (**Figures 2D–I**). Often the cells in islands are arranged in a swirl-like pattern (**Figure 2H**). Islands can be highlighted by a halo-like neuron-poor zone or capsule (Goldman-Rakic, 1982). These were seen particularly at 1 day and 1 month of age (**Figures 2G,H**). The cell density in islands was higher than in the surrounding matrix territory at 1 day and 12 months of age (**Figure 2L**), consistent with previous estimates (Goldman-Rakic, 1982). The cell densities in both compartments significantly decreased during the first postnatal year, possibly reflecting the increased striatal volume (**Figure 1B**) and selective programmed cell death of neurons. Apoptotic profiles representing developmental programmed cell death were seen prominently in the postnatal rhesus monkey striatum (**Figure 2J**). The identification of these cells as apoptotic was based on criteria such as cellular shrinkage and separation from the surrounding neuropil to appear as a haloed object and, importantly, condensation of the nucleus into dark round masses (**Figure 2J**) as described before (Al-Abdulla et al., 1998; Martin et al., 1999; Northington et al., 2001; Lok and Martin, 2002). In the caudate nucleus most of the apoptotic profiles were found usually in the matrix (**Figure 2K**), while in the putamen apoptotic profiles were distributed nearly equivalently in striosome and matrix compartments (**Figure 2L**).

### POSTNATAL MATURATION OF THE RHESUS MONKEY DORSAL STRIATAL MOSAIC

#### Overview

**FIGURE 3:**
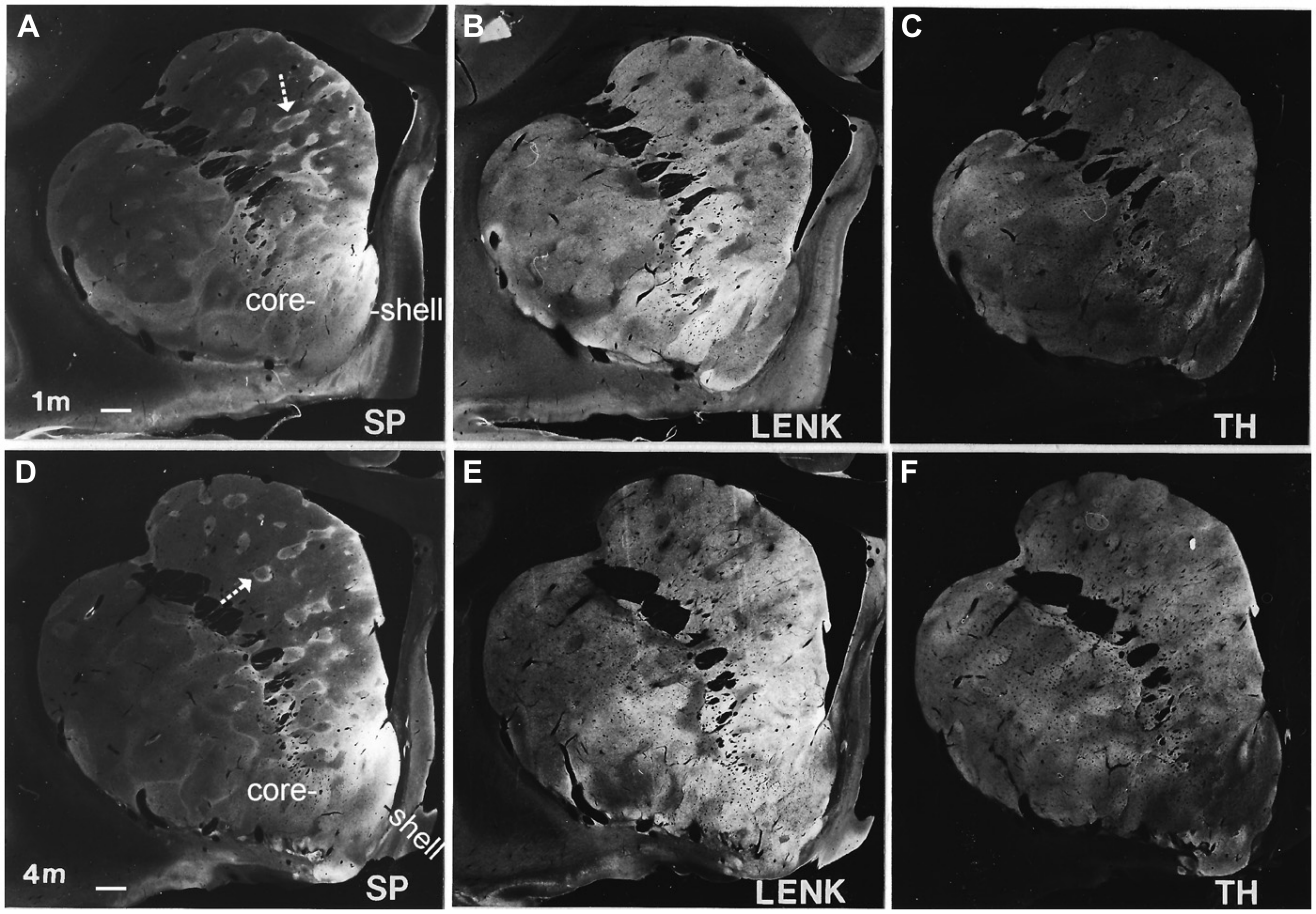
**Localization of substance P (SP, A,D), leucine-enkephalin (LENK, B,E), and TH, (C,F) in the anterior striatum of rhesus monkeys at 1 and 4 months of age.** Images were created by using the immunoperoxidase/DAB-stained hemisections as a photographic “negative” and printing on to photographic paper to generate macrographs. White-gray represents immunoreactivity with white being the highest intensity and gray lower intensity. Black is no immunoreactivity. Arrows identify SP immunopositive striosomes with center-low and surround-high organization. Right is medial. Top is dorsal. The nucleus accumbens of the ventral striatum is divided into shell and core regions. Scale bar = 750 μm.

The striatum in infant rhesus monkeys underwent prominent qualitative and quantitative remodeling in its chemical neuroanatomy during the first year of postnatal life (**Figures 3** and **5–8**). Early postnatally the striatum contained immunoreactivity for several neurotransmitter and neuronal markers that were also present in the striatum of mature rhesus monkeys (e.g., TH, SP, LENK, and calbindin), but the spatial locations (**Figures 3**, **5**, and **8**) and quantities (**Figures 6** and **7**) of immunoreactivities associated with neurons and neuropil changed as a function of age. Preadsorption control experiments showed that an excess of neuropeptide antigen abolished all immunoreactivity detected in the infant striatum with antibodies to TH, LENK, SP, and calbindin (data not shown), and both monoclonal and polyclonal antibodies to TH, SP, and LENK yielded similar maturational patterns (data not shown).

#### Postnatal day one and 1 month of age

**FIGURE 4:**
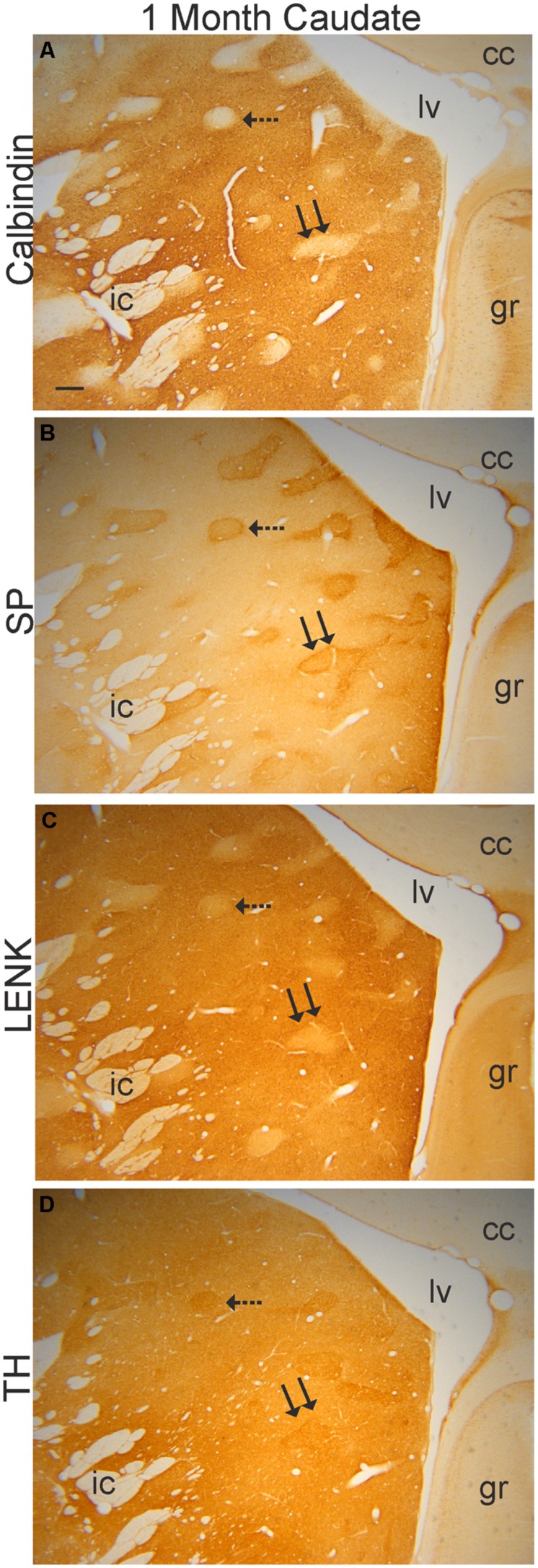
**Verification of the mosaic compartmentation of the caudate nucleus in a 1-month-old rhesus monkey.** Adjacent sections (40 μm in thickness) were stained immunohistochemically with antibodies to calbindin **(A)**, SP **(B)**, LENK **(C)**, and TH **(D)** using DAB as the chromogen. Immunoreactivity appears as brown staining. Hatched and double arrows identify two different striosomes that are present in each section. Right is medial. Top is dorsal. cc, corpus callosum; gr, gyrus rectus; ic, internal capsule; lv, lateral ventricle. Scale bar in **(A)** = 300 μm **(B–D)**.

**FIGURE 5:**
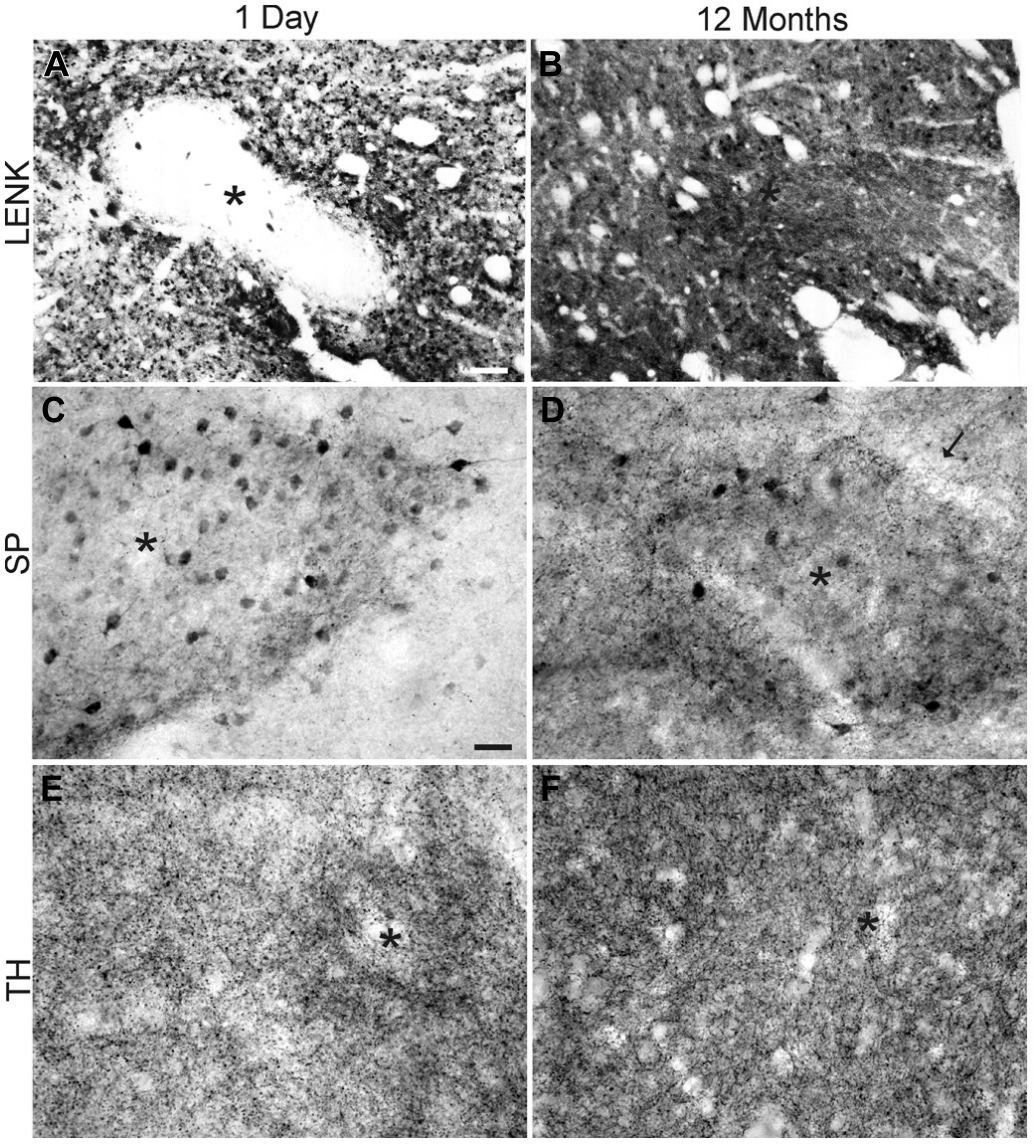
**Comparison of the cellular localizations of LENK, SP, and tyrosine hydroxylase (TH) immunoreactivity in the dorsomedial caudate nucleus of the rhesus monkeys at 1 day (A,C,E) and 12 months (B,D,F) of age. (A)** LENK immunoreactivity is very low in striosomes (asterisk) at 1 day of age, while, in the matrix, numerous immunopositive neurons are present but matrix neuropil has low to moderate immunoreactivity. **(B)** LENK immunoreactivity is enriched in striosomes (asterisk) at 12 months, and the surrounding matrix neuropil has high immunoreactivity. **(C)** Striosomes (asterisk) in 1-day-old monkeys contain numerous SP-immunoreactive neuronal cell bodies and the striosomal neuropil is enriched in SP-immunoreactive puncta, while the surrounding matrix has low SP immunoreactivity. **(D)** The matrix in 12-month-old monkeys is enriched in SP-immunoreactive puncta, while the striosomes (asterisk) are still distinct from the matrix because of SP-immmunoreactive neuronal cell bodies and greater neuropil immunoreactivity. Arrow identifies annular border of striosome. **(E)** Striosomes (asterisk) at 1 day of age have higher TH immunoreactivity (representing dopaminergic innervation) than the surrounding matrix. **(F)** The matrix at 12 months of age has greater TH immunoreactivity than striosomes (asterisk). Scale bars = 175 μm **(A,** same for **B)**, 35 μm **(C**, same for **D–F)**.

**FIGURE 6:**
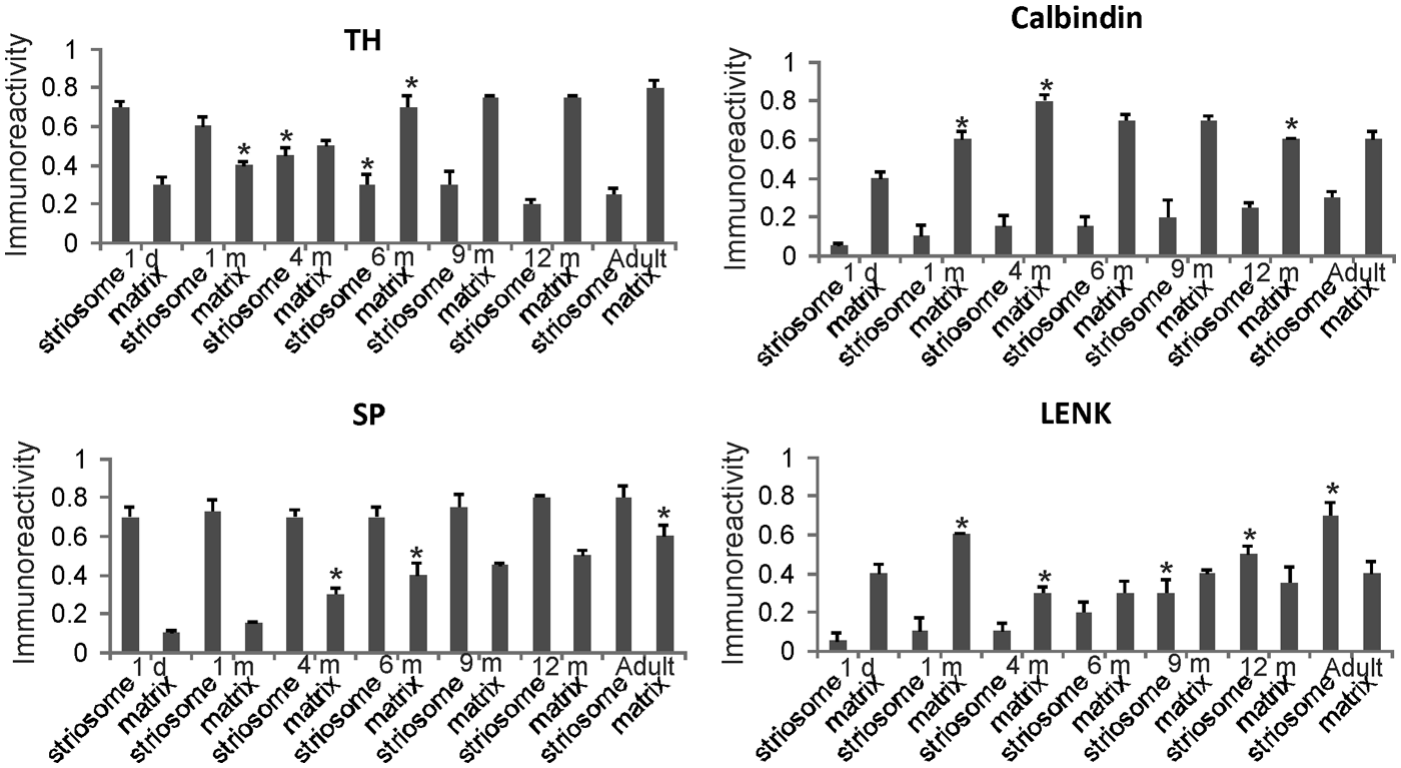
**Densitometric measurements of TH, calbindin, SP, and LENK immunoreactivities in the dorsomedial caudate nucleus of rhesus monkeys at 1 day, 1, 4, 6, 9, and 12 months of age and in adults (4–7 years).** Values are mean ± SD of relative optical density. Significant differences (higher or lower) identified by asterisks in the following comparisons. TH: 1 m matrix vs. 1 d matrix, *p* < 0.05 higher; 4 m striosome vs. 1 m striosome, *p* < 0.05 lower; 6 m striosome vs. 4 m striosome, *p* < 0.05 lower; 6 m matrix vs. 4 m matrix, *p* < 0.01 higher. Calbindin: 1 m matrix vs. 1 d matrix, *p* < 0.01 higher; 1 m matrix vs. 1 m matrix, *p* < 0.05 higher; 12 m matrix vs. 9 m matrix, *p* < 0.05 lower. SP: 4 m matrix vs. 1 m matrix, *p* < 0.05 higher; 6 m matrix vs. 4 m matrix, *p* < 0.05 higher; adult matrix vs. 12 m matrix, *p* < 0.05 higher. LENK: 1 m matrix vs. 1 d matrix, *p* < 0.01 higher; 4 m matrix vs. 1 m matrix, *p* < 0.01 lower; 9 m striosome vs. 6 m striosome, *p* < 0.05 higher; 12 m striosome vs. 9 m striosome, *p* < 0.05 higher; adult striosome vs. 12 m striosome, *p* < 0.01 higher.

**FIGURE 7:**
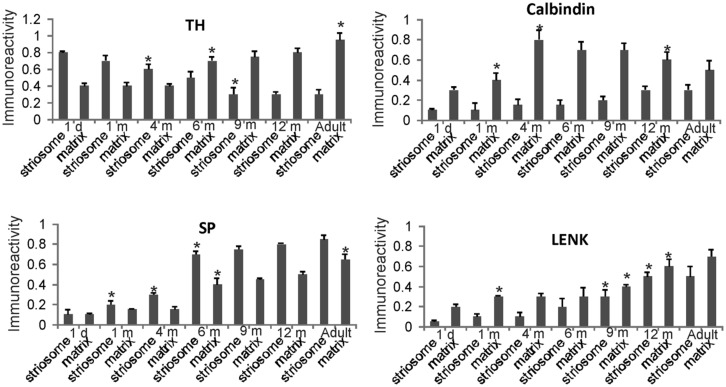
**Densitometric measurements of TH, calbindin, SP, and LENK immunoreactivities in the central putamen of rhesus monkeys at 1 day, 1, 4, 6, 9, and 12 months of age and in adults (4–7 years).** Values are mean ± SD of relative optical density. Significant differences (higher or lower) identified by asterisks in the following comparisons. TH: 4 and 6 m striosome vs. 1 d striosome, *p* < 0.01 lower; 6 m matrix vs. 1 d, 1 m, 4 m matrix, *p* < 0.01 higher; 9 m striosome vs. 6 m striosome, *p* < 0.05 lower; adult matrix vs. 12 m matrix, *p* < 0.05 higher. Calbindin: 1 m matrix vs. 1 d matrix, *p* < 0.05 higher; 4 m matrix vs. 1 m matrix, *p* < 0.01 higher; 12 m matrix vs. 4, 6, 9 m matrix, *p* < 0.05 lower. SP: 1 m striosome vs. 1 d striosome, *p* < 0.05 higher; 4 m striosome vs. 1 m striosome, *p* < 0.05 higher; 6 m striosome vs. 4 m striosome, *p* < 0.01 higher; 6 m matrix vs. 4 m matrix, *p* < 0.05 higher; adult matrix vs. 12 m matrix, *p* < 0.05 higher. LENK: 1 m matrix vs. 1 d matrix, *p* < 0.05 higher; 9 m striosome vs. 6 m striosome, *p* < 0.05; 9 m matrix vs. 6 m matrix, *p* < 0.05; 12 m striosome vs. 9 m striosome, *p* < 0.05 higher; 12 m matrix vs. 9 m matrix *p* < 0.01 higher.

Early postnatally throughout the anterior–posterior extent of the caudate nucleus and putamen, islands enriched in TH immunoreactivity, originating putatively from nigrostriatal and mesolimbic dopaminergic neurons (Loizou, 1972; Tennyson et al., 1972; Graybiel and Ragsdale, 1983; Zahm et al., 1990; Heimer and Van Hoesen, 2006; Haber et al., 2012), were prominent against a matrix territory containing less TH immunoreactivity than in the islands (**Figures 3C,F**). At postnatal day one and at 1 month of age, the distributions of LENK and SP immunoreactivities within the striatum were also compartmental (**Figures 3A,B**). The specific identities of the striosomal and matrix compartments were verified by the compartmental distribution of calbindin immunoreactivity (**Figures 3** and **4**). The presence of calbindin immunoreactivity defines matrix neurons in monkey (Cote et al., 1991). In newborn and 1-month-old rhesus monkey striatum, the distribution of calbindin immunostaining was similar to LENK and was highly enriched in the matrix neurons in the caudate (**Figures 4A,C** and **5A**). At birth most neurons within the matrix of the caudate nucleus and putamen were LENK-immunoreactive (**Figure 5A**), while striosomes were very apparent at this age because they were mostly devoid of LENK immunoreactivity (**Figures 3B**, **4C**, **5A**, **6**, and **7**) but were enriched in SP immunoreactivity (**Figures 3A**, **4B**, **6**, and **7**). The SP-low matrix in the caudate nucleus and putamen contrasted sharply with the SP-high striosomes (**Figures 3A**, **4B**, **6**, and **7**). The SP immunoreactivity within striosomes was localized to many neuronal cell bodies and puncta within the neuropil (**Figure 5C**), while the matrix compartment had few SP immunoreactive neurons and low SP immunoreactivity in the neuropil (**Figures 4B** and **5C**). During the first postnatal month, LENK-poor and SP-enriched striosomes showed a one-to-one spatial alignment with the TH-positive striosomes (**Figures 3A–C** and **4B–D**). SP-positive striosomes often had a subcompartmental appearance with a center-low and a rim surround-high level of immunoreactivity distribution (**Figures 3A** and **4B**).

Quantitatively the levels of immunoreactivity for TH, SP, LENK, and calbindin in the caudate nucleus (**Figure 6**) and putamen (**Figure 7**) in 1-day-old rhesus monkeys were significantly different from 12-month-old and adult monkeys. TH immunoreactivity was significantly higher in striosomes and lower in matrix at 1 day compared 12-month-old monkeys in both caudate and putamen (**Figures 6** and **7**). At 1 day compared to 12 months, SP immunoreactivity was significantly lower in the caudate matrix (**Figure 6**), and lower in both the caudate and putamen striosomes and matrix (**Figures 6** and **7**). Comparison of the same ages revealed that LENK immunoreactivity was significantly lower in caudate striosomes (**Figure 6**) and in putamen striosomes and matrix (**Figure 7**) early in life compared to mature ages. During maturation from 1 day of age to 1 month of age, the caudate matrix had significant increases in TH, calbindin, and LENK immunoreactivities (**Figure 6**), and the putamen matrix had significant increases in calbindin and LENK immunoreactivities (**Figure 7**). At the same time, SP immunoreactivity in putamen striosomes increased significantly (**Figure 7**).

#### Postnatal month four

The density of TH-positive immunoreactivity was greater within the caudate and putamen matrix compartment of 4-month-old monkeys compared to younger monkeys (**Figures 3F**, **6**, and **7**). With the greater enrichment of TH immunoreactivity in the matrix, the visualization of many discrete striosomes became obscured in the caudate (**Figure 3F**). Opposite changes in the patterns of LENK and SP immunoreactivities continued to be observed at 4 months of age. Relative to earlier postnatal periods, the level of LENK immunoreactivity was reduced significantly in the matrix compartment of the dorsal lateral caudate and putamen (**Figure 6**). In contrast, the caudate and putamen of monkeys at 4 months of age showed significantly increased SP and calbindin immunoreactivities in the matrix compared to younger ages (**Figures 6** and **7**).

#### Postnatal month six and nine

**FIGURE 8:**
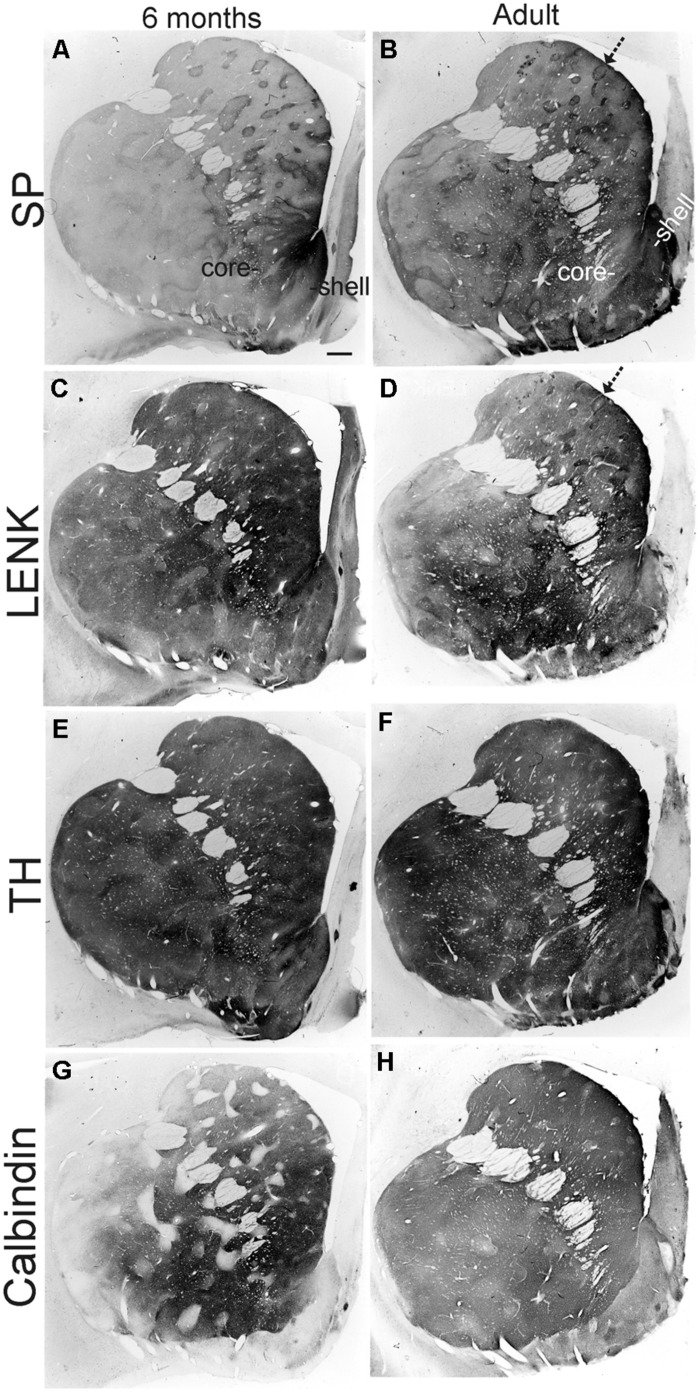
**Localizations of SP, (A,B), LENK, **(C,D)**, TH, **(E,F)**, and calbindin **(G,H)** immunoreactivity in the anterior striatum of rhesus monkeys at 6 months of age and in adulthood (7 years).** Images are macrophotographs of immunoperoxidase/DAB-stained sections. Immunoreactivity is black-gray signal. White in the image is no immunoreactivity. The localization patterns for SP, LENK, TH, and calbindin all undergo salient changes between 6 months of age and adulthood. Arrows identify striosomes. Right is medial. Top is dorsal. The nucleus accumbens of the ventral striatum is divided into shell and core regions. Scale bar = 750 μm.

The highlight of the developing rhesus monkey striatum at 6 months of age was the significant increase in TH immunoreactivity in the matrix compartment (**Figures 6**, **7**, and **8E**) that now exceeded the level of TH immunoreactivity in the striosomes, thus completing the inversion in the striosome-matrix pattern that was seen at birth (**Figures 6** and **7**). At this age the general pattern of LENK immunoreactivity-poor striosomes and LENK immunoreactivity-enriched matrix was sustained (**Figures 6**, **7**, and **8C**), while SP immunoreactivity in the matrix increased significantly (**Figures 6**, **7**, and **8A**). Calbindin was still a matrix marker at this time, except for in the lateral putamen where calbindin levels were low compared to adults (**Figures 8G,H**). The levels of immunoreactivity for TH, SP, LENK, and calbindin were similar and 6 and 9 months of age (**Figures 6** and **7**).

#### Postnatal month 12

In 1-year-old monkeys, striatal chemoarchitecture was similar qualitatively to the patterns seen in caudate nucleus and putamen in 9-month-old monkeys, but there were some nuanced quantitative differences. Compared to 9 months, calbindin immunoreactivity significantly decreased in the caudate matrix, and LENK immunoreactivity increased in caudate striosomes (**Figure 6**). In putamen, calbindin immunoreactivity decreased significantly in the matrix, while LENK immunoreactivity increased significantly in the striosomal and matrix compartments (**Figure 7**).

#### Adult

The chemical neuroanatomy of the striatum in adult rhesus monkeys has been studied previously (Haber and Elde, 1982; Graybiel and Ragsdale, 1983; Martin et al., 1991a,b). SP, ENK, TH, and calbindin immunoreactivities are localized differentially within the striatum, and the general distributions of these antigens in the dorsal striatum of individual monkeys are consistent among colony reared animals (Martin et al., 1991b). As in previous studies (Haber and Elde, 1982; Graybiel and Ragsdale, 1983; Martin et al., 1991a,b), the adult rhesus monkey striatum was divisible into striosomal and matrix compartments (**Figures 8B,D–F**). The intensity of TH immunoreactivity was lower in striosomes than in the surrounding matrix, because the matrix was more highly enriched in TH immunoreactive fibers and terminals; in addition, the density of calbindin-immunoreactive neuronal cell bodies was greater in the matrix than in striosomes (**Figures 6** and **7**). SP and ENK-immunoreactive striosomes occurred throughout the striatum and corresponded (1:1) with zones lower in TH and calbindin immunoreactivities (**Figures 8B,D–H**).

Comparison of 12-month-old monkeys and adults revealed that, while the patterned ingrowth of dopaminergic nigrostriatal afferents seen as TH immunoreactivity was qualitatively complete, some measurable differences were discerned (**Figures 6** and **7**). The matrix of adult putamen had higher levels if TH immunoreactivity and SP immunoreactivity compared to monkeys at 12 months (**Figure 7**). In caudate of adult monkeys, SP immunoreactivity was greater in the matrix and LENK immunoreactivity was greater in striosomes compared to 1-year-old monkeys (**Figure 6**).

### POSTNATAL MATURATION OF THE RHESUS MONKEY NUCLEUS ACCUMBENS

**FIGURE 9:**
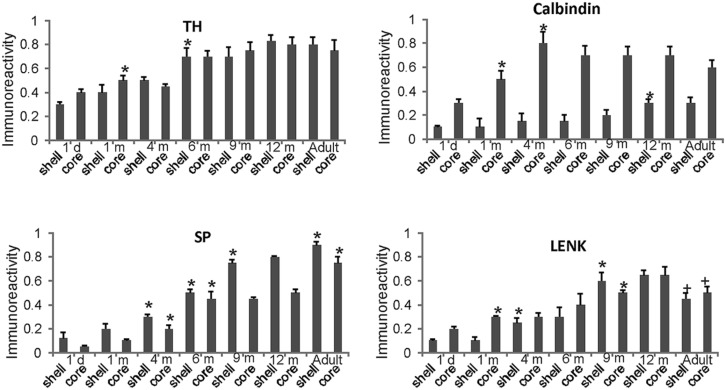
**Densitometric measurements of TH, calbindin, SP, and LENK immunoreactivities in the nucleus accumben (shell and core regions) of rhesus monkeys at 1 day, 1, 4, 6, 9, and 12 months of age and in adults (4–7 years).** Values are mean ± SD of relative optical density. Significant differences (higher or lower) identified by symbols (* or +) in the following comparisons. TH: 1 m core vs. 1 d core, *p* < 0.01 higher; 6 m vs. 4 m shell *p* < 0.001 higher. Calbindin: 1 m core vs. 1 d core, *p* < 0.001 higher; 4 m core vs. 1 m core, *p* < 0.01 higher. 12 m shell vs. 9 m shell, *p* < 0.05 higher. SP: 4 m shell and core vs. 2 m shell and core, *p* < 0.01 higher; 6 m shell and core vs. 4 m shell and core, *p* < 0.001 higher; 9 m shell vs. 6 m shell, *p* < 0.001 higher; adult shell and core vs. 12 m shell and core, *p* < 0.01 higher. LENK: 1 m core vs. 1 d core, *p* < 0.01 higher; 4 vs. 1 m shell, *p* < 0.01 higher; 9 m shell vs. 6 m shell, *p* < 0.05 higher; 9 m core vs. 6 m core, *p* < 0.05 higher; adult shell vs. 12 shell, *p* < 0.01, lower; adult core vs. 12 core, *p* < 0.05, lower.

The nucleus accumbens in primates is differentiated into shell and core regions (Meredith et al., 1996; Heimer et al., 1997; Brauer et al., 2000), and these regions are clearly divisible at an early age (**Figure 3**). At one day to 1 month of age, the accumbens shell was divisible from the core by the low level of LENK immunoreactivity (**Figures 3B** and **9**), while the dorsal part of the shell was enriched in SP immunoreactivity (**Figures 3A** and **9**). At 4 months of age, the shell gained LENK immunoreactivity (**Figures 3E** and **9**), and the level of SP immunoreactivity increased (**Figures 3D** and **9**). Surprisingly, at birth through 4 months of age the levels of TH immunoreactivity in the nucleus accumbens were low, except at the interface between the shell and core regions (**Figure 3C**). At 6 months of age the localization patterns for SP and LENK immunoreactivities (**Figures 8A,C**) were similar to those seen at 4 months (**Figures 3D,E**), but the overall levels of SP immunoreactivity was increased compared to the earlier age (**Figure 9**). The level of TH immunoreactivty in the nucleus accumben was sharply increased throughout the shell and core at 6 months (**Figures 8E** and **9**) and at 12 months compared to 4 months (**Figure 3F**), while the patterns of calbindin immunoreactivity clearly delineated the shell from the core (**Figure 8G**). The core was high in calbindin immunoreactivity and the shell was low in calbindin immunoreactivity (**Figure 9**). Between 12 months of age and adulthood, the salient changes to maturity were a near complete occupation of the shell with SP immunoreactivity (**Figures 8B** and **9**), a decline in LENK immunoreactivity in core and shell regions (**Figures 8D** and **9**), and an increase in shell calbindin immunoreactivity, but still remaining distinctly lower in calbindin immunoreactivity than the core (**Figures 8H** and **9**).

## DISCUSSION

The major contribution of this study is the demonstration that the rhesus monkey striatum has a prolonged postnatal maturation extending over a year. This maturation was reflected by changes in cytology and chemical neuroanatomy. In many instances the remodeling was dramatic and involved reversal of chemical patterns in the different compartments of the striatum. In general, several gradual developmental events were observed in the maturing striatum, including reductions in cellular density, increased innervation by dopaminergic afferents, an initial high expression of LENK (in matrix) followed by a subsequent down-regulation of ENK expression, and a progressive increase in SP expression (in matrix). Developmental gradients were apparent in the dorsal-ventral, medial–lateral, and anterior–posterior axes of the striatum.

The development of the striatum is an example of neural pattern formation (i.e., how groups of cells assemble, form appropriate patterns of connectivity, and establish their neurotransmitter/receptor phenotypes to form functional neuronal ensembles) because it is divided into different cellular compartments. The cellular compartments were discernible even with basic histological staining methods. The Nissl-identified cellular islands in the caudate nucleus of rhesus monkey have been shown before in fetal monkeys (Goldman-Rakic, 1981) and in 2-month-old, 1-year-old, and adult monkeys (Goldman-Rakic, 1981, 1982). They are not entirely equivalent to striosomes (Graybiel and Ragsdale, 1978) because the cellular islands are fewer in number than histochemically- and immunohistochemically identified striosomes (Selemon et al., 1994), but many Nissl-identified cellular islands register with striosomes (Selemon et al., 1994). Goldman-Rakic (1981) also showed elegantly that in infant rhesus monkeys the cellular islands are largely free of prefrontostriatal innervation, while the surrounding matrix has dense innervation from frontal cortex. We also observed a subcompartmental organization within striosomes, prominently in the caudate nucleus. Subsets of SP- and LENK-immunoreactive striosomes possessed center-low and surround-high levels of immunoreactivity. This subcompartmental organization of striosomes has been seen before in rhesus monkeys by visualizing SP and SP receptor immunoreactivity (Jakab et al., 1996) and in humans by detecting neurotensin receptor binding (Faull et al., 1989), SP immunoreactivity (Holt et al., 1997), LENK immunoreactivity (Manley et al., 1994; Prensa et al., 1999), and limbic system-associated membrane protein immunoreactivity (Prensa et al., 1999).

The neurogenesis in the primate striatum has been nicely documented in the distant and recent literature. Studies in rhesus monkey fetus show that neostriatal neurons are generated during a period lasting about 50 days (gestational days 36–85 of a 165 day gestation) with maximal neuronal proliferations occurring at about fetal day 45 (Brand and Rakic, 1979, 1980). The human striatum emerges embryonically from the lateral ganglionic eminence (Pauly et al., 2014) with the caudate being identified at the 13 mm stage (~6.5 weeks) and the putamen recognized at the 26 mm stage (~8.5 weeks; Humphrey, 1968). Human striatal neurons originate from PAX6-negative lateral ganglionic eminence precursors, and the immature striatal neurons express the postmitotic marker FOXP1 already at 50 days post-fertilization (Pauly et al., 2014). Limited neurogenesis persists in adult striatum of squirrel monkeys (Bedard et al., 2002) and humans (Ernst et al., 2014). Interestingly, the adult generated striatal neurons in humans are interneurons (Ernst et al., 2014), but in squirrel monkeys they are projection neurons rather than interneurons (Bedard et al., 2006).

Cells within striosomes and matrix appear to have distinct developmental programs, because neurons within different compartments of the neostriatal mosaic are generated at different times during gestation. Neurons that reside within striosomes become postmitotic before neurons that reside within the matrix in rat (Marchand and Lajoie, 1986; van der Kooy and Fishell, 1987). The striatum in rhesus monkey appears to follow a similar histogenetic pattern regarding the differential birth of striosome and matrix neurons (Brand and Rakic, 1979). In addition, the establishment of efferent connections of early born striatal neurons with midbrain targets may allay developmental cell death within the rodent neostriatum (Fishell and van der Kooy, 1991). However, the developmental events that underlie striatal cell fate and the formation and maintenance of neuronal phenotype expression and afferent/efferent connectivity of distinct populations of striatal neurons are unclear. Important questions about striatal development concern the relative contributions of genetic, extrinsic epigenetic, and environmental determinants in the generation of its compartmentalization (Fishell and van der Kooy, 1987; van der Kooy and Fishell, 1987; Martin et al., 1991b). These questions apply to the prenatal stages of compartment formation and to the postnatal period when the striatum undergoes extensive maturational changes for an extended duration during infancy as shown here. Gender might also be an important variable in this regard, but because of our small sample size, gender differences were not examined. The present study shows, for the first time, that at birth the rhesus monkey striatum has an immature cytologic and chemoarchitectonic organization and that the postnatal maturation of the striatum is accompanied by prominent temporal and spatial changes in the expression of several neuronal phenotypes.

Experimental data on the development of the chemical neuroanatomy of the non-human primate striatum is sparse, despite the prominent anatomical representation of the striatum in primates and its functional role in complex motor and non-motor behaviors including sensorimotor integration, motor control, cognition, and social-emotional behaviors such as mood and addiction, and its role in several human neurological disorders (Crittenden and Graybiel, 2011; Baez-Mendoza and Schultz, 2013). A few available studies of the striatum in baboons show prenatal and/or postnatal changes in adrenergic, cholinergic, and dopaminergic ligand binding sites by autoradiography (Slesinger et al., 1988; Lowenstein et al., 1989) as well as sodium-dependent high affinity choline uptake binding sites (Lowenstein et al., 1987). However the normal spatiotemporal development of connectively, cell adhesion molecules, synapses, neurotransmitters, receptor proteins, and signal transduction molecules in the striatum of non-human primates is mostly unknown. One study used infant rhesus monkeys at 2, 8, 10, and 31 days of age to show that mRNA expression of forkhead motif transcription factor gene FOXP2 is enriched in striosomes and that expression declines with maturation (Takahashi et al., 2008). We demonstrate in this study three new ideas regarding the primate striatum. First, the mature anatomical organization of the dorsal and ventral striatum in primate is established postnatally, because the cellular and chemoarchitectonic differentiation of the dorsal striatum in rhesus monkey continues significantly throughout the first postnatal year. Second, striatal maturation is topographically ordered, because the putamen, caudate nucleus, and nucleus accumbens reach adult-like chemoarchitecture at different times. This idea is best illustrated by comparing the graphs of TH, LENK, and calbindin immunoreactivities for caudate (**Figure 6**), putamen (**Figure 7**), and nucleus accumbens (**Figure 9**). The major developmental increases in TH are skewed to the left for caudate and to the right for putamen and the major increases in LENK in putamen occur later than in caudate. The maturation of the nucleus accumbens has a maturation tempo similar to caudate nucleus for TH, Calbindin, and LENK. Third, striosomes and matrix compartments mature postnatally at different rates. Based on these results, we conclude that primate striatum undergoes marked prolonged remodeling during postnatal development and speculate that development of normal functions within the striatum occurs postnatally and that the normal maturation of primate striatum is contingent on multiple cellular and molecular events that are specified genetically and epigenetically by environmental factors. This developmental theory of striatal postnatal maturation would be in line with the finding that striatal organization is profoundly abnormal in isolation-reared rhesus monkeys presenting with severe psychopathology (Martin et al., 1991b).

The striatum of non-primates (mostly, rat, cat, and rabbit) also is immature at birth and undergoes changes postnatally in organization (Graybiel et al., 1981). Thus like primates, the mature organization of the striosomes-matrix pattern in adult rodents, rabbits, and cats is established postnatally, but the rate of morphological and neurochemical development is very accelerated in non-primates compared to primates. In non-primates, the expressions of many neuronal and non-neuronal molecular phenotypes are developmentally regulated within the striatum. For example, in the developing non-primate striatum, ontogenetic changes occur in: the levels of enzymatic activities for acetylcholinesterase, TH, glutamic acid decarboxylase, and choline acetyltransferase (McGeer et al., 1971); the histochemical localizations of acetylcholinesterase (Graybiel and Ragsdale, 1980; Graybiel et al., 1981) and dopamine (Loizou, 1972; Olson et al., 1972; Tennyson et al., 1972; van der Kooy, 1984); the immunocytochemical localizations of SP, enkephalin, neurotensin, TH (Graybiel et al., 1981; Goedert et al., 1985; Boylan et al., 1990; Zahm et al., 1990); the expression of mRNA for neurotransmitter synthesis enzymes (Greif et al., 1992) and primary transcripts for neurotransmitters (Tecott et al., 1989; Haverstick et al., 1990; Cimino et al., 1991); the autoradiographic localizations of binding sites for SP (Quirion and Dam, 1986), opiates (Kent et al., 1981; van der Kooy, 1984), dopamine (Murrin and Zeng, 1989), acetylcholine (Nastuk and Graybiel, 1985), and other neuropeptides not normally abundant in adult striatum (e.g., oxytocin and vasopressin; Petracca et al., 1986; Tribollet et al., 1989); the expression of genes that encode messenger RNA for dopamine receptors (Guennoun and Bloch, 1991; Mack et al., 1991); and glial-derived extracellular matrix glycoproteins (Steindler et al., 1988). Importantly, it is thought that the striatum in cat is adult-like by postnatal day 21 (Graybiel and Ragsdale, 1983), and the rodent striatum has a similar developmental timing with maturity of SP and neurotensin patterns achieved at about postnatal day 20 (Zahm et al., 1990). However, a recent study of the mesocorticolimbic system in rat revealed a protracted postnatal maturation (Yetnikoff et al., 2014). The comparatively rapid maturation of the striatum in non-primates contrasts with our results showing that the duration of maturation of the rhesus monkey striatum extends to 12 months of age and beyond. Thus the developmental timing of cellular differentiation of the striatum is extraordinarily different among primates and non-primates. The retention of more immature patterns of chemoarchitecture for longer periods of time during postnatal development (neoteny) may result in a greater vulnerability of basal ganglia organization to experience and environmental stressors in infants and toddlers.

Because of the uniqueness of this animal cohort and the general lack of information on basic morphometric parameters in developing rhesus monkeys, we measured brain weights and striatal volumes. Our findings were very informative. The ratio of brain weight to body weight showed a steep progressive decline early in life, while a postnatal change in striatal volume was modest. The early postnatal decrease in brain weight to body weight ratio in rhesus monkeys sharply contrasts with observations in human infants. With humans there is a rapid rise until about 2 years of age followed by a slow gradual decline until about 13–15 years of age (Dekaban, 1978). Recent MRI studies also reveal major differences in the postnatal maturation of gray matter to white matter ratios in infant rhesus monkeys and humans (Sakai et al., 2012). Infant chimpanzees are also very distinct from infant rhesus monkeys in the maturation of gray matter and white matter, but chimpanzees have some similarities to human infants (Sakai et al., 2012). We also report that striatal volume increases postnatally in rhesus monkeys. The finding is interesting because caudate volume determined by MRI was shown typically to decrease with child development, but in autistic children caudate volume increases in association with the presence of repetitive behaviors (Langen et al., 2009). These distinctions in the postnatal development of monkey, ape, and human brains have important relevance to the evolutionary biology of the brain (Sakai et al., 2012) and may be possibly relevant to the vulnerability of the higher primate brain to early childhood environmental stress (Bogart et al., 2014). Recent imaging studies have shown that early life stress, such as traumatic adverse childhood events, is associated with reduced caudate volume in adults (Cohen et al., 2006) and reduced nucleus accumbens activation and adolescent depression (Goff et al., 2013).

We also studied aspects of striatal cytology during postnatal development. Neuronal density in striatum was found to decline during the first year of life. This reduction of cell density occurred in matrix and striosome compartments. It may be related to the modest increase in striatal volume that was shown to occur during this time. More likely, the loss of neuronal density could also be related to the elimination of cells through normal developmental programmed cell death (Martin, 2001). We found prominent evidence for naturally occurring programmed cell death through apoptosis in the striatum of infant monkeys. We used Nissl staining to identify apoptotic cells. This approach is simple, but reliable, and we have shown that cells with a Nissl-staining pattern of apoptosis are usually positive for TUNEL and cleaved caspase-3 (Al-Abdulla et al., 1998; Martin et al., 1999; Northington et al., 2001; Lok and Martin, 2002; Mueller et al., 2005). Apoptosis that is detected by Nissl staining also registers with internucleosomal fragmentation of DNA detected by Southern blotting (Lok and Martin, 2002). This apoptosis data is useful because it demonstrates that another component of the postnatal remodeling of the monkey striatum involves cell death, in addition to the changes in neuropeptide localization and abundance.

Our current work is likely to be relevant to human infants because it may be reflective of the neoteny of the higher primate brain. It is known that the human prefrontal cortex undergoes extensive synaptic remodeling at puberty and beyond adolescence (Petanjek et al., 2011). The adult human dorsal striatum has a prominent striosome-matrix organization, as well as additional characteristics that could be peculiar to the human striatum (Holt et al., 1997; Prensa et al., 1999). The adult human nucleus accumbens also has a fascinating complexity with features not seen in monkeys (Prensa et al., 2003). Moreover, babies that are born prematurely show MRI abnormalities in the ventral striatum (Shi et al., 2013) and abnormalities in frontostriatal connectivity (Duerden et al., 2013). The ontogeny of the human striatum has not been studied comprehensively, and, indeed, very few studies have been done. Most studies to date have focused on the fetal brain (13–40 weeks gestation) and have shown in striatum patchy distributions with striosomal enrichments of acetylcholinesterase (AChE) and muscarinic cholinergic binding sites (Nastuk and Graybiel, 1985), AChE and NADPH-diaphorase (Sajin et al., 1992), calbindin (Letinic and Kostovic, 1996), dopamine D1 receptor and dynorphin mRNA (Brana et al., 1996), glutamate receptor scaffold kinase anchoring protein (Ulfig et al., 2001), and synaptophysin (Ulfig et al., 2000). A study of postnatal human brain revealed an inversion from an immature pattern of calbindin-enriched striosomes to a mature pattern of calbindin-enriched matrix sometime between term gestation to 18 months of life (Letinic and Kostovic, 1996). Another study showed that the immature pattern of AChE-enriched patches is still present at 3 months of age (Graybiel and Ragsdale, 1980). Thus, the human striatum also undergoes major prenatal and postnatal remodeling in its chemical neuroanatomy, but the extent is unknown, begging the question of whether the postnatal pattern formation and neurogenesis in this brain region, known to function in complex behaviors and shown to be involved in several motor and behavioral disorders (Crittenden and Graybiel, 2011; Baez-Mendoza and Schultz, 2013), is vulnerable to environmental influences and stress through epigenetic mechanisms.

## AUTHOR CONTRIBUTIONS

Conceived and designed experiments: Linda C. Cork and Lee J. Martin. Performed experiments: Lee J. Martin (with technical assistance). Analyzed data: Lee J. Martin (with technical assistance). Wrote paper: Lee J. Martin.

## Conflict of Interest Statement

The authors declare that the research was conducted in the absence of any commercial or financial relationships that could be construed as a potential conflict of interest.
